# The Emergence of AI in Public Health Is Calling for Operational Ethics to Foster Responsible Uses

**DOI:** 10.3390/ijerph22040568

**Published:** 2025-04-04

**Authors:** Gauthier Chassang, Jérôme Béranger, Emmanuelle Rial-Sebbag

**Affiliations:** 1 CERPOP, Université de Toulouse, Inserm, UPS, 31000 Toulouse, France; jeromeberanger@hotmail.com (J.B.); emmanuelle.rial@univ-tlse3.fr (E.R.-S.); 2 Genotoul Societal Platform, Ethics and Biosciences, GIS Genotoul Occitanie, 31000 Toulouse, France; 3 Unesco Chair, Ethics Science and Society (E2S), Working Group on Digital Ethics, 31000 Toulouse, France

**Keywords:** public health ethics, artificial intelligence, responsible AI, AI ethics, law, international standards, international law, public health law

## Abstract

This paper discusses the responsible use of artificial intelligence (AI) in public health and in medicine, and questions the development of AI ethics in international guidelines from a public health perspective. How can a global ethics approach help conceive responsible AI development and use for improving public health? By analysing key international guidelines in AI ethics (UNESCO, WHO, European High-Level Expert Group on AI) and the available literature, this paper advocates conceiving proper ethical and legal frameworks and implementation tools for AI in public health, based on a pragmatic risk-based approach. It highlights how ethical AI principles meet public health objectives and focuses on their value by addressing the meaning of human-centred innovations, transparency, accountability, diversity, equity, privacy protection, technical robustness, environmental protection, and post-marketing surveillance. It concludes that AI technology can reconcile individual and collective ethical approaches to public health, but requires specific legal frameworks and interdisciplinary efforts. Prospects include the development of supporting data infrastructures, of stakeholders’ involvement to ensure long-term commitment and trust, of the public’s and users’ education, and of international organisations’ capacity to coordinate and monitor AI developments. It formulates a proposal to reflect on an integrated transparent public health functionality in digital applications processing data.

## 1. Introduction

Artificial Intelligence (AI) systems (AIS) are promising tools that are widely developing in the fields of health, (bio)medicine, and public health. AI has a strong transformative effect for health systems and should not be seen as an end but as means to achieve strategic priorities and Sustainable Development Goals [1,2]. In order to succeed in providing its full potential to enhance global public health coverage, to efficiently address health disparities, and to fully protect individuals’ and populations’ dignity, stakeholders in AI design, development, and use should implement respect for ethics and human rights. Worldwide, the specific ethical and legal framing of these new technologies is still in the early stages of development. Public health applications will necessitate specific attention as the use of AI might bring as many benefits as risks for policy-makers and populations concerned by a public health policy or intervention.

In this paper we adopt a broad notion of “public health” as defined by the World Health Organisation (WHO). The WHO International Health Regulations (IHR) define public health as a domain covering any type of “illness or medical condition, irrespective of origin or source, that presents or could present significant harm to humans” [3]. This approach encompasses any health hazard including biological threats (e.g., infectious disease outbreaks), chemical events, radio-nuclear incidents, food safety issues, and zoonotic diseases. The core purpose of public health actions is to prevent, protect against, and control public health risks, and to provide a response to daily health issues of the population and to the international spread of diseases. In this regard, the IHR provide key obligations for countries to develop core capacities to detect, assess, report public health data, and respond to potential emergencies. It establishes a global governance mechanism to report any risk of international concern and to coordinate adapted response. It also offers WHO support to countries to develop and maintain preparedness to public health crisis management. In the European Union (EU), public health protection is a shared responsibility between the EU and its Member States (MS). The EU does not define nor directly manage public health policies but complement national policies and support cooperation between MS. The EU intends to protect a high level of public health through its policies and actions [4] (art. 168). It supports MS in addressing communicable and non-communicable diseases, cross-border health threats, promoting health systems’ sustainability, reducing health inequalities, and promoting research in public health areas. This includes EU initiatives for improving healthcare systems, healthier lifestyles, digital health, and the fight against antimicrobial resistance while guaranteeing fundamental rights. Moreover, the EU supports and protects public health through regulations, ethical guidelines, funding programs (such as the EU4Health [5]), and partnerships [6]. Research and technological innovations in AI are now regulated in the EU by the Artificial Intelligence Act [7] (AIA), a horizontal regulation that is not focused on health technologies but liaises with other important regulations for the field such as the EU Medical Device Regulations (MDR) [8] and the General Data Protection Regulation (GDPR) [9]. The AIA defines AIS as “a machine-based system that is designed to operate with varying levels of autonomy and that may exhibit adaptiveness after deployment, and that, for explicit or implicit objectives, infers, from the input it receives, how to generate outputs such as predictions, content, recommendations, or decisions that can influence physical or virtual environments” [7] (art. 3(1)). Regarding public health, AIS will essentially be used as predictive technologies based on big data analytic tools intended to support decision-making and interventions. These cover a wide range of software technologies, from expert to generative AIS, and several other applications in public health. The spreading of AIS in healthcare and in public health requires ethical reflections to ensure its responsible development and use. How would a global ethical approach help to conceptualise the responsible use of AI to improve public health?

In this paper, we first provide a concise summary of the similarities and differentiation between clinical and public health ethics to consider in shaping a global ethical approach to AIS (Section 3.1). Then, we provide an overview of AI applications based on their purpose of use in public health, and of their expected benefits and potential risks (Section 3.2). We identify and analyse key principles stated by different ethical norms for envisaging responsible development and use of AIS in public health at international and EU level. Where relevant, we refer to legal developments and existing binding rules incorporating ethical principles in order to illustrate part of the operationalisation of ethics in public health (Section 3.3). We question their interoperability and completeness to identify strengths and potential weaknesses to address for building a global comprehensive and adapted ethical framework for AI in public health that could either feed further legal developments or enlighten public health practices (Section 4).

## 2. Materials and Methods

We performed a literature review through PubMed and Google Scholar to identify relevant publications. We used combinations of free-text keywords (Artificial Intelligence or AI, and public health, and ethics or international law or regulation or guidelines or legal or ethical principles). This search was limited to peer-reviewed journal articles and book chapters published between 2010 and 2025 to capture 1/the landscape of AI in public health practice and 2/to embrace the most recent ethical and regulatory frameworks. This search has been complemented by additional resources like books, studies, and reports from international organisations, websites information, and recent regulatory documents addressing AI and health at International and European Union (EU) levels, including some illustrative EU Member States regulatory sources (e.g., France), which have been published from 2001. These documents include soft-law instruments (Ethical guidelines, Authoritative norms) and hard-law instruments (Treaties, EU Regulatory instruments) fixing rules that impact the development, deployment, and use of AIS in public health. We performed a careful selection of the results based on titles and abstracts screening by using the following inclusion criteria: the relevance of the topic addressed regarding AI ethics in public health, the release date of the documents (publications after 2010 were favoured), the source (peer-reviewed journals, book chapters), and the language (English or French only). We excluded publications unrelated to ethical or legal issues affecting public health and non-peer-reviewed journals or opinions. Full-text screening was performed on shortlisted studies, with discrepancies resolved through discussion. Then, we performed a comparative analysis of the selected documents through the lens of international public health law and normative ethics, we addressed their content in order to envisage the complementarity of current approaches regarding AI, ethics, and law in public health, and drew a picture of ongoing works, remaining issues, and complexities to advance the field while promoting the population’s health.

## 3. Results

### 3.1. Ethics in Public Health and Ethics in Clinical Care or Biomedical Research: Common Principles, Specific Implementation

The ethical framework in clinical care and research is based on biomedical ethics establishing the principles of respect for individual Autonomy, Beneficence, Non-maleficence, and Justice [10]. These principles are widely used in these contexts but they can present limits to guide concrete decision-making in digital innovation and public health action due to specific ethical tensions or dilemmas [11]. If they are always considered in public health actions, in recent decades, public health ethics has tended to become more autonomous, with specific developments. The identification of appropriate public health values and principles began in the research setting in 1991 with the adoption of the International Ethical Guidelines for Epidemiological Studies by the Council for International Organisations of Medical Sciences (CIOMS) which were revised in 2009 [12]. These guidelines, largely inspired from the Beauchamps and Childress principles [10], emphasised the specificity of public health research ethics when it comes to “the unit of study (which) is not an individual person but a community or other group”. The background paragraph of the 2009 Guidelines clearly highlighted that overarching bioethical principles are common to biomedical research ethics and to public health research ethics. This led to a major update of the CIOMS research guidelines in 2016 with the adoption of the revised “International ethical guidelines for health-related research involving humans” [13] which covers both types of research. CIOMS emphasised that, regardless of the type of the research, “there is no clear distinction between the ethics of social science research, behavioural studies, public health surveillance and the ethics of other research activities. The current scope (of the guidelines) is confined to the classic activities that fall under health-related research with humans, such as observational research, clinical trials, biobanking and epidemiological studies”. As a result, the guidelines address the most common principles such as consent or data use, while defining the specificities of public health research ethics. Thus, regarding implementation in Public Health, professionals proposed the Principles of the Ethical Practice of Public Health, endorsed by the American Public Health Association [14] in 2002 and revised in 2019 [15]. It identifies specific values necessary for responsible actions: Professionalism and Trust, Health and Safety, Health Justice and Equity, Interdependence and Solidarity, Human Rights and Civil Liberties, Inclusivity and Engagement. Currently, the development of such codes is encouraged [16], as well as dedicated tools to ethically assess policies and interventions in public health [17].

In order to implement an adequate framework for AI ethics in the context of public health that will adequately support contributions to human well-being and human rights, it is important to consider existing differences and shared characteristics of each framework.

The main differences between clinical practice and public health stems from their respective objects of study and purposes, from who is affected, from and who decides and implements interventions [18]. In terms of scope, whereas clinical ethics focuses on the specific needs, issues, and interests of identifiable individuals and patients, public health ethics examines health issues and needs at a population level. The latter is made up of heterogeneous groups of individuals from a variety of ethnic, social, cultural, economic, and religious backgrounds and environments. In terms of purposes, biomedical ethics applies to specific patient cases, focusing on personalised care and treatment decisions in clinical settings or on protective measures for participants in research settings, while public health ethics seeks to promote the health and well-being of the population as a whole. It applies ethical principles to broader public health interventions, such as vaccination programs or health education campaigns, which affect entire populations. Public health ethics is characterised by the complexity of its issues: it is not limited to the well-being and health of individuals, but must also promote equity and defend the health interests of all groups, particularly those from marginalised, oppressed or disadvantaged communities [19]. Stakeholders involved are also different. Biomedical ethics involve healthcare providers and individual patients in a clinical context or investigators and participants in a research context, with decisions often made in one-on-one settings. Public health ethics involves a wider range of stakeholders, including policymakers, community leaders, and the general public. Decisions are often made at a policy level, in collective settings, and involving different processes [11].

These differences are a source of ethical tensions. Public health ethical issues often involve trade-offs between individual and collective interests, such as between equity, social justice, and individual freedom [20], the health of an individual being considered in a broader populational context involving considerations for the health of third individuals. These trade-offs are likely becoming increasingly common and complex in the field of AI. Nevertheless, AI technologies have the potential to drastically approximate individual and population levels to facilitate and improve health management and interventions.

The development of adequate recommendations, and the challenges of identifying the best values to frame public health and research ethics, have set the stage for better defining the key challenges and for building a future framework for the responsible use of AI in public health. Those recommendations are particularly important for building efficient guarantees and operational systems allowing populations data collection and processing capabilities while explicating human rights protection [21]. In this regard, the law is an essential enabler of the preparedness approach in public health to protect and enhance population health [22].

### 3.2. The Rise of AI Applications in Public Health, Potential Benefits and Risks

The recent and progressive introduction of AIS in medical practice [23] presents advantages for public health in terms of individual health and well-being improvement [24]. AIS applications in public health, whether or not based on generative AI, could significantly improve analytical performance and related processes and practices from the perspective of health promotion (See Appendix A.1, Table A1). While AIS increase the capacity to collect, organise, share, and analyse individual health data, they also raise questions about how these individual results translate into population health, and what ethical rules and regulatory interactions apply. In order to maximise the added value of AIS introduced in clinical and public health sectors, work remains to be done in order to align the primary collection of data from individuals and their use for public health purposes. This necessitates the development of specific, interoperable, and networked technological systems to gather relevant data, within or outside health systems settings, for training AI models tailored to public health purposes. There are still important challenges to ensure the smooth integration of AIS into clinical settings [25] and to structure repositories of FAIR data (Findable, Available, Interoperable, and Reusable) [26] for public health purposes. Strategic priorities for modernising health systems in view of the digital or AI transformation of public health are being prepared at international and national levels. A recent study [27] of such plans and recommendations at international, Canadian, United States, and United Kingdom levels identified six priorities for the successful integration and use of AIS in public health organisations. These priorities aim to address needs regarding contemporary data governance, investment in modernising data and analytic infrastructure and procedures, skills gaps in the workforce, the development of strategic collaborative partnerships, the use of good AI practices for transparency [28] and reproducibility, and, finally, the explicit consideration of equity and bias. While we remain at the onset of the development and deployment of these new technologies in public health, the potential to improving population health is as important as economical, ethical, legal, and strategic interests from private actors and governments in the domain, i.e., huge. These various interests should not be underestimated as they can undermine the ethical approach to technological developments and deployment. The most recent introduction of AIS in public health during the COVID-19 pandemic is illustrative of the important questions raised concerning the respect and potential restrictions of individuals freedoms for public health protection purposes. The risks associated with AIS in public health are numerous and vary according to their use cases. They fall into several categories, each reflecting specific technical, ethical, and social issues (See Appendix A.2, Table A2).

According to the WHO [29], the increasing use of generative AIS based on large multimodal models (LMMs), including large language models (LLMs), poses unique risks in healthcare that might adversely affect public health activities. These risks stem from LMMs’ abilities to process diverse data inputs, such as text, images, and videos, and generate various creative outputs to mimic human communication and to accomplish tasks without being explicitly programmed for them. Generative AIS require special attention as they have potentials to improve healthcare, public health research, interventions, and health education as decision-support tools [30]. These potentials rely on their abilities to process big heterogeneous datasets in real-time and on their ease of use (chatbots). Today, these AIS are still a general-purpose technology. However, Generative AI is based on complex neural networks and deep learning models (foundation models) which are known to be opaque, increasing the risk of undetected bias, of unfair and inequitable outcomes, and reducing the human ability to understand the AI’s decision-making process. The large amount of diverse data used to train the foundation models (that can include omics data, biosensors, clinical, social, and environmental data), as well as the variety of input data that can be entered by users, amplify risks related to sensitive data confidentiality and intellectual property. Generative AIS are also known for their tendency to produce significantly different outputs depending on the prompt used to query them (challenging their apparent ease of use) and to hallucinate (the model generates incorrect or misleading results). Outputs can be convincingly presented to users who may not be fully exercising their critical thinking. This includes risks of deskilling and overconfidence that could lead to erroneous, unduly oriented, or harmful decisions. The quality of generated data could therefore not be sufficient for professional uses, neither for healthcare nor for public health uses. These specificities introduce complexities beyond those associated with traditional, unimodal AIS. The ethics of deep learning in clinical settings is poorly studied; application to public health is a step further. Nevertheless, the uniqueness of generative AI and LMMs opens new avenues for the creation of tailored AIS able to efficiently exploit large public health datasets and improve processes to ethically promote population health.

Ethical and regulatory reflections must inform and guide AI technological developments and uses. Framing AI as an emerging technology is presently on the agenda of international and national organisations dealing with ethics of new technologies and legislation in order to identify values and regulatory issues at stake when developing, deploying, and using AI in several contexts.

### 3.3. The Building of Relevant Ethical Norms for Responsible AI in Public Health

International guidelines governing AI are still developing through a principlism approach.

An analysis of the most recent ethical guidelines adopted at international and European levels for framing AI developments is illustrative of how individual and collective interests are more than ever connected and considered as a whole when addressing AI technologies, although they do not focus on public health. These ethical rules should be embraced by innovators in order to ensure that innovation meaningfully serves the public interest and common good. A recent scoping review [31] highlights key themes addressed by the literature on ethical and legal principles for AI in public health. These are specifically related to AIS safety, privacy, health equity, bias, confidentiality, transparency, accountability, and social justice, with various degrees of coverage. In this paper, we propose a complementary analysis that concentrates on selected official international documents adopted by representative organisations (specialising in science and technology with UNESCO; health with the WHO; and AI with the EU expert group), allowing us to provide an in-depth legal and ethical analysis for building accurate norms for AI in Public Health.

Thus, the key international frameworks considered here are the UNESCO Recommendation on the Ethics of AI [32], the World Health Organisation (WHO) Guidelines on AI [29,33], and the European High-Level Expert Group (HLEG) Guidelines on Trustworthy AI [34]. All offer valuable insights into the ethical application of AI, particularly in public health, and considered relevant pre-existing works at an international level, such as the OECD recommendations.

In terms of focus areas, the WHO Guidelines are specific to health and well-being. They formulate tailored recommendations for using AI in clinical care, public health, and research. The UNESCO recommendation was the first global AI ethics document and addresses the broader societal implications of AI, emphasising cultural diversity and international co-operation in AI governance. The European HLEG Guidelines were adopted before the EU Regulation on AI and inspired it. They focus on fostering innovation in Europe while ensuring trustworthiness in AI across all domains by reference to EU values and fundamental rights [35].

In terms of addressees, the WHO and UNESCO documents are primarily addressed to governments and policy-makers. They provide a global multilateral perspective on AI ethics with more or fewer details on implementation frameworks. The HLEG Guidelines are addressed first to AI researchers and developers in the EU.

In terms of ethical dimensions, the WHO explores health-specific dilemmas, like prioritising AI solutions in low-resource settings. UNESCO highlights the ethical challenges of capacity-building in diverse socio-economic contexts and the effects of AI on cultural heritage and development. The organisation provides a Readiness Assessment Methodology (RAM) “to help countries understand how prepared they are to apply AI ethically and responsibly for all their citizens” [36]. It also provides a framework for the Ethical Impact Assessments of AI projects [37]. The HLEG focuses on fostering trust in AI by adhering to the EU values and provide a structured framework for assessing AI trustworthiness through principles like technical robustness with a specific self-assessment tool for developers [38]. They built on the above-mentioned four principles of biomedical ethics to derive specific principles for AIS.

Therefore, these examples provide a comprehensive ethical coverage in the domain, even if non-exhaustive, combining considerations of global applicability of ethical principles while addressing localised, cultural, socio-economic, and sector-specific challenges.

An analysis of these documents allows us to highlight common ethical principles that should drive innovation for a responsible AI development and use, including in the public health sector as we will illustrate, and that can be backed by law or further regulations.

Our comparative analysis highlights the following nine ethical principles that should be specifically addressed and implemented in public health policies or interventions:

**1. Ethics-by-design** [32] (points 34, 65–66, 72, 95, 108), [33] (pp. 65–69), [34] (p. 21) also referred to as “design for values” [33] (pp. 65–69), [32] (pp. 18–20). This principle both asks governments to enact laws and regulation that will comply with and support the development of ethical requirements and human rights standards, with investments in AI-specialised monitoring agencies and independent third parties’ control, and requires that developers address ethical and legal issues that their system may raise in the early stage of the project building. The “design for values” paradigm aims at integrating, within the design of technologies, efficient and transparent elements allowing the respect of human dignity, freedom, equality, and solidarity along the AIS lifecycle. This cautious approach requires us to be proactive in assessing, iteratively, the impact of the AIS and in diminishing risks (at the design, development, deployment, post-deployment monitoring, pre-decommission phases) compared to a pre-existing set of objectives and ethical requirements. Ethics-by-design in AI calls for establishing the roots for responsibilities regimes attached to the States and AIS providers. Practical assessment tools have been proposed to achieve this process. In particular, in relation to the HLEG Guidelines on Trustworthy AI, the ALTAI checklist and web tool [38] helps AI providers to self-assess the ethics of their system. The European Commission, together with the literature [39], proposes complementary systematic approaches [40], and some National Health Agencies provide guidelines for integrating ethics during data collection and pre-processing, and the building and evaluation of the AI algorithm [41]. In addition, since 2024, the EU AIA makes it mandatory for the deployer of high-risk AIS to perform a prior-to-deployment Fundamental Right Impact Assessment (FRIA) [7] (art. 27, rec. 96) of the AIS in its own context, based on the documentation made available by the AIS provider and on the intended use of the AIS. While the EU does not provide specific tools for performing this specific assessment, it is possible to use the HUDERIA methodology (Human Rights, Democracy and Rule of Law Impact Assessment) [42], adopted in connection with the Council of Europe Convention on AI, which necessitates to be used in consideration of the context in which the system is or will be deployed and used. At international level, the Z-inspection process [43] can be used for a variety of compliance checks depending on the applicable ethical framework [44]. These tools allow, based on a list of ethical pre-conditions, to assess the likelihood and severity of potential risks that may arise before, during and after the AIS deployment. Through the assessment phases, ethical tensions and potential dilemmas [45] can arise that will need to be identified, addressed and documented. The iterative assessment process serves to maintain ethical reflection around the AIS and to continuously align the provider’s claims and system’s evolutions with the evolutions of technical and ethical standards as well as other societal requirements and expectations, in order to maintain trustworthiness.

**2. Human-Centric Approach** to AIS developments. All emphasise the importance of AI systems being designed and implemented to enhance human well-being, dignity, and rights, in the public interest [33] (p. 26). In public health, for example, AI should aim to improve health outcomes and well-being for society as a whole. This can include using AI for early disease detection, optimising resource allocation in healthcare, or managing public health crises. The principle implies a notion of human empowerment as AIS should enhance human decision-making and human autonomy [46], (point 1.1) [34]. In public health, AI can provide decision support to healthcare professionals, ensuring they have better data and insights to make informed decisions [47]. It also encapsulates the need for ensuring human oversight, requiring to design and implement mechanisms for human surveillance and control of AIS operations in order to ensure that AI processes and outputs can be reviewed and overridden by human experts. Implement mechanisms for human oversight, ensuring AI recommendations in public health can be reviewed and overridden by human experts. For instance, diagnostic AIS should include the option for healthcare providers to validate or reject AI-generated diagnoses or recommendations. The same should be possible for public health professionals and citizens dealing with recommendations based on predictions from machine-learning models. Aside from the need to work on effective and user-friendly human-machine interfaces, decision-trees and options for human decision-making, transparency of the system and models will be key to allow enlightened and responsible human action.

**3. Transparency and Accountability** call for mechanisms that ensure clarity in AI decision-making process in order to ensure public trust [32] (principles 37–40, 44–45, Policy Areas 2, 8, 9), [33] (pp. 26–28), (points 1.4, 1.7) [34]. Transparency includes to set up AI governance policies detailing efficient information processes for patients and citizens that are concerned by the use of their personal information [33] (p. 87, Figure 2) or that will be affected by the AIS outputs, to attribute roles to competent professionals in the use and decision-making processes based on AIS, to design transparent algorithms and provide intelligible outputs to AIS users, but it also covers challenges related to the explainability of machine-learning models and their acceptability in specific domains [33] (pp. 106–108). AIS should provide understandable explanations of their decisions [46,48,49]. Transparency includes elaborating clear documentation on how AI models are developed, trained, and validated as well as clear instructions of use [7] (Rec. 72, art. 13). Interpretability of the output requires specific works. Users should be able to understand the data and variables underlying the output so that they can use their expert knowledge to exercise critical appraisal and explain the principles of functioning of the AIS to a third. These details should always be made available under request. Explainability is particularly useful for allowing AIS auditability and for checking compliance with ethical, legal, and technical standards. This could include external reviews and certifications. Traceability shall ensure that the processes and data used by the AIS in each operation can be identified from the origin, sourced, so that their role in the formulation of the output can be clarified. Generally, transparency measures through information campaigns and education programs intended to explain the rationale and practical aspects of an AIS development or use with adapted informational content depending on the targeted audience, context and role in the public health process are very important in an age of public defiance against institutions and technologies. This effort aims to clearly present the adopted public health ethics approach in AI development, justifications for AIS selection and conditions of use in a specific context, to allow public debate, to answer potential fears and eventually make a collaborative decision on the use of AIS. Accountability frameworks are also crucial [32] (Principles 42–43, Policy Area 2, points 55, 69), especially in life-impacting domains like health and should clearly establish responsibility chains along the AIS lifecycle. AI literacy is of major importance for ensuring that all users and persons in charge of monitoring the system’s operations will take the best advantage of these technologies while being enabled to act knowingly, ethically and responsibly. Any public health intervention must be evidence-based, and decision-makers shall be liable and able to explain the process they followed to reach conclusions according to the state-of-the-art. Therefore, any public health institution envisaging to adopt or to advise AIS should be prepared. Accountability requires to set up clear responsibility regimes along the AI value chain [50], from the AIS provider to the end-user. In public health, this means defining who is responsible for AI-driven decisions and ensuring that there are mechanisms for addressing any issues that arise, including legal pathways to access justice in case of damage claims. In this regard the EU revised its rules on liability regarding defective products in light of the challenges brought by AIS on the effectiveness of individual rights protection [51]. In addition, public authorities should perform transparent self-assessment of existing and proposed AIS including the assessment of whether the adoption of an AIS is appropriate in a given context and if this would result in violations or abuses of human rights. In such a case they shall be able to prohibit this use [32] (Policy Area 2, point 57).

**4. Diversity, Equity, Fairness and Non-discrimination**, which have strong echoes in public health ethics, are also central to AI ethics for ensuring that AIS do not exacerbate existing inequalities [32] (Principles 28–30, Policy Area 3), [33] (pp. 28–29), [34] (point 1.5). Data quality is essential. Therefore, researchers developing public health applications must actively counteract bias in health and medical datasets used to train the AI models [52]. This aims at ensuring, during the development and throughout the deployment and AIS lifecycle, that the model or foreseeable misuses of the device do not perpetuate or amplify biases that result in errors and potential disadvantages or damages (e.g., where AIS is used for health resource allocation and prioritisation) [33] (pp. 49–50). This involves using diverse datasets and regularly testing cycles for bias detection and mitigation in public health applications. This package of interlaced principles also covers the ethical concept of inclusive design. This concept aims at ensuring that the AIS consider the diverse needs of all populations, particularly vulnerable groups, ensuring equitable access to the technology, adaptive uses where feasible, and improved access to AI-based healthcare innovations [52]. The use of online services users’ content, such as social networks data, poses several challenges in terms of individual rights but also in terms of data quality. The spreading of false and misleading content online, notably through generative AI, leads to an unprecedented “informational pollution”. Strategies to watermark AI-generated content [53] are developing to help identifying synthetic content and apply certain authenticity checks before use [54], such as with the C2PA content credentials mark (CR) [55], but certain technical challenges remain to ensure their permanency and non-falsifiability [56].

In public health, equitable access to the necessary technology to prevent, diagnose and treat diseases is still a challenge. Ownership and intellectual property rights applicable to public health AIS may be ethically criticised where they limit or exclude entire categories of persons, countries or territories, with limited resources, from the benefit of their uses [33] (pp. 93–95). Therefore, in this domain, these principles also call for the development of open source AIS. Open source AIS can be defined as a system made available under terms and in a way that grants the freedoms to use the system for any purpose and without having to ask for permission, to study how the system works and inspect its components, to modify the system for any purpose, including to change its output, to share the system for others to use with or without modifications, for any purpose [57]. This Open Source AI Definition (OSAID) does not impede the application of protective legislations that may apply to certain types of testing and training data, for example privacy or intellectual property laws. Advocating for developing open source AIS in public health could facilitate their global uptake, community review and improve public health benefit sharing.

**5. Privacy, data protection and inclusive governance** are particularly highlighted [33], (Principles 32–34, Policy Area 3) [32], (point 1.3) [34]. Maintaining individuals’ data confidentiality [58,59] and data minimisation, only using necessary data for their functions [60], while using large-scale health data for AI training and AI-driven insights is constitutive of responsible innovation in AI and public trust. The ethical challenges mainly relate to the rules for accessing relevant data about health determinants, both for innovating and for the operations of the AIS once made available on the market, without compromising individuals’ identity and privacy [61]. Public health AIS may necessitate constant access to real-world data, including in real-time, to perform its operations but also to gather data for training the model, what raises issues such as individuals’ consent practice, pseudonymisation or anonymisation techniques and data confidentiality management. Privacy challenges exist both in clinical AI [62] and public health AI and calls for certain trade-offs to ensure AIS performance and public engagement. During the COVID-19 pandemic, a large number of mass surveillance devices and related databases were developed and used to fight against the virus spreading and facilitate public health interventions. Contact tracing apps have been a perfect example of the ethical tensions arising from a digital technological solution for public health emergencies filling the gap between individual and population health needs through data analysis. Despite the pressing needs to face public health threats caused by the rapid spreading and evolutions of the virus coupled with the overload and lack of resources of health systems in most countries of the world, privacy concerns have not been put aside, particularly in Europe [63,64,65], where the GDPR offered a “ready-made functional blueprint for system design that is compatible with fundamental rights” [66]. The use of these apps remained voluntary, temporary (data were collected for a limited period of time), publicly monitored, and implemented data minimisation, encryption and other privacy-enhancing technologies maximising their potential for adoption by the public. They provided different levels of information to users including contact tracing (bluetooth-based) and tracking (GPS-based) but also in some cases symptom check and self-diagnosis information, trustworthy public health news, self-managed support tool for homebound diagnosed patients and support from medical staffs in the form of follows-up on homebound patients, among others [67]. Throughout the pandemic, unprecedented efforts have been made, involving public and private entities, to link public health data analysis with daily individual’s life and to design appropriate real-world data processing pipelines and models (centralised or decentralised) [68] benefiting individual and collective interests, while striking a balance between privacy and effectiveness [69]. For instance, an ethical trade-off between data minimisation and the performance of public health algorithms necessitating minimising biases can base on the collection of equitable data from all population [62]. Contact tracing apps provided important epidemiological insights [70,71,72] and demonstrated the potential and limits of AI to enhance public health responses [73,74].

Outside the pandemic’s context, access to individual health data remains a challenge to develop public health AIS. Even where laws exist, such as in the EU with the GDPR, the access to high quality, representative and sufficiently precise health data for training the models and building algorithms is still challenging. While health data access and sharing can always be legitimately restricted or prevented by law, it is more often enabled by law when justified by public health purposes and provided that adequate organisational and technical safeguards are ensured depending on the data processing nature, purposes, risks and potential effects on data subjects. In the EU, the GDPR offers several legal grounds to process personal health data, genetic, biometric or other types of sensitive data where necessary to achieve a substantial public interest [9] (art. 9(2)(g)); or for archiving purposes in the public interest, scientific or historical research purposes or statistical purposes [9] (art. 9(2)(j)), with potential national legal exceptions to a number of individual rights [9] (art. 89(2)). In case of pandemic for example, a processing can be legally grounded, even without individual consent, if “necessary for reasons of public interest in the area of public health, such as protecting against serious cross-border threats to health or ensuring high standards of quality and safety of health care and of medicinal products or medical devices, on the basis of Union or Member State law which provides for suitable and specific measures to safeguard the rights and freedoms of the data subject, in particular professional secrecy” [9] (art. 9(2)(i)). Despite these provisions favourable to data sharing, public health researchers still experience difficulties in practice where lock-in approaches to health data continue to apply or where legal uncertainties remain (e.g., for accessing precise socio-professional data or geolocalisation in the study of health determinants). Other issues concern the lack of dataset quality or completeness to perform certain AI studies [75] and the lack of data sharing infrastructure. In the EU, this challenge is tackled through the building of the European Science Cloud (EOSC) developing FAIR data platforms and services [76] and with the European Health Data Space (EHDS). The latter will organise a decentralised infrastructure relying on national Health Data Access Bodies (HDAB) for accessing health data, whether personal or non-personal data, based on an obligation of data holders to provide access to the data they possess and necessary metadata to feed a catalogue of available data for research uses. As the EU Regulation on the EHDS (EHDSR) [77] reminds, “the COVID-19 pandemic has highlighted the imperative of having timely access to quality electronic health data for health threats preparedness and response, as well as for prevention, diagnosis and treatment and secondary use of health data” [77] (rec. 2). The EHDS builds on the GDPR to improve access to and control by data subjects over their personal electronic health data for healthcare purposes (primary use) and to better achieve other purposes that would benefit society, such as research, innovation, policy-making, health threats preparedness and response, including to prevent and address future pandemics, to develop personalised medicine or official statistics (secondary use) [77] (rec. 3–5). Data access for secondary uses for the purposes listed, and under the conditions fixed, by the EHDSR are based on data subject’s informed opt-out [77] (art. 53–54, Chapter IV). States’ duties to organise and facilitate access to relevant, high-quality data for public health research are detailed and strong safeguards for personal data are consistently highlighted, in particular with the setting up of secured processing environments. The text includes measures to facilitate health data access for training AI and measures for coordinating the EHDS with the recognised data altruism organisations created under the EU Data Governance Act (DGA) [78] whose objective is to increase data availability while overcoming technical barriers to sharing and reuse in the public interest.

In France for example, the Health Data Hub (HDH), the future EHDS HDAB, organises and structures access to most of the health databases on the French territory, notably through the National Health Data Platform (SNDS) that makes available anonymised and, where relevant, pseudonymised health data to researchers. Access is subject to prior ethics approval of the projects where required by law and to strict privacy protection measures. The HDH acts in close collaboration with the French data protection authority (Commission Nationale Informatique et Libertés—CNIL) that authorises personal data processing. The HDH offers services and updated tools to help researchers understand the data, their organisation, bias and potential limits, and help the opening of research data for further reuses (open access or open data). The HDH maintains an open library of algorithms [79] in health that can be used to better exploit the data if relevant for the project purpose.

Generally, there is a need to conceive collective governance mechanisms of health data for public health purposes. The EHDS project is a good example in this regard and could support federated data learning practices [33] (pp. 86–87) preserving data sovereignty while performing controlled data visitation without moving analysed data [80], unlocking the data potential for AI development in public health. The structuration and regulation of data intermediaries and data altruism organisations in the EU, in particular with the DGA, can support different models of data sharing (i.e., personal information management systems, data cooperatives, data trusts, data unions, data marketplaces, and data sharing pools) [81]. Some of them support collective governance models, collaborative decision-making on data commons and production of collective benefits or public value. The creation of collective data rights based on a chain of trustworthy relationships from the data subject to the data holder and to the data user deserves further attention in the context of public health AI development. This includes addressing the question of consent and potential ethical and lawful alternatives [32] (pp. 81–86) in the context of health data sharing [82] and AI data visitation [83]. The UNESCO identifies data cooperatives as a relevant governance structure for maintaining data sovereignty by indigenous communities and other marginalised groups [33] (p. 90).

AIS used for public health surveillance, including prediction-based surveillance, outbreak responses and emergency preparedness through the use of a variety of data, including digital traces, are legally inscribing those data as part of health data, by destination. Health data collection activities such as web scraping and data mining for building an AI training database raise particular ethical issues for individual rights and privacy. In the European context, data controllers practicing web scraping and data mining are responsible for complying with the GDPR and applicable laws and should work closely with their Data Protection Officer to assess and plan measures of privacy compliance, in particular to ensure lawfulness of the processing, data minimisation, quality and integrity, transparency and pathways for data subjects rights exercise (in particular for them to oppose to the processing). In this regard the French CNIL provides some guidance to facilitate compliance and opens the possibility for developers to rely on the legal basis of legitimate interest [84]. This guidance includes, for example, a recommendation to limit data collection to freely accessible data (i.e., content accessible to any user not registered on the site in question and without creating an account) and manifestly made public by users having explicitly expressed their choice beforehand to make their data accessible to an unlimited number of people in order to ensure that they do not lose control of the data they publish online (excluding, for example, data published by individuals on social networks for private use, such as information contained in private profiles or groups). For other types of private sensitive data, it should be recommended to contact the website’s responsible entity to design appropriate and transparent data collection protocols. The CNIL envisages creating a public registry of data controllers practicing web scraping, including for AI development, with a link towards their privacy policy, information about the modalities for the exercise of data subject’s rights and, facultatively, a listing of scraped websites. The inscription in this registry would facilitate transparency of the processing without presuming the lawfulness of the processing undertaken.

Finally, interesting developments around synthetic data [85] generation and digital twins could provide solutions to improve access to relevant health data that keeps scientific informational value while avoiding restrictions related to privacy laws. Digital twin technologies seem particularly interesting for public health purposes and might take advantages of ongoing developments in healthcare [86] and other connected infrastructures, in particular with dynamic self-adaptive digital twins. These having the capacity to acquire real-time data and update their model require a digital thread keeping track of evolution and communication with the real-world object, system, or organism, they could mimic complex phenomenon at population scale. Current projects develop digital twins of smart cities that can be used to perform in silico studies simulating both the impact of public health events, such as epidemic, on infrastructures, and the impact of public health policy decisions and interventions [e.g., [87]]. The challenge is to access qualitative data to forge a truly representative digital twin.

**6. Evidence-based development, technical robustness and post-marketing monitoring** call for a step-by-step approach to AIS spreading in public health based on scientific evidence gathered through experimentation. AIS in public health must be reliable [88] and secure [89], ensuring patient data is protected and the system functions accurately under various conditions. Robust technologies require strong cybersecurity measures and regular audits. Health technology assessment and control by public and independent authorities is crucial. The development of technical standards is also very much needed, in particular for AIS that will not be considered as medical devices falling under the corresponding legal framework and potential certification scheme. Experiments, in-lab or in real-life conditions, serve to evaluate and validate the AIS assets, including the training datasets, and to test the operational functioning and use of the device [90]. In public health, this requires to validate both the statistical validity, the public health utility and the economic utility of the AIS. Accuracy and error management will necessitate elaborate AI models minimising unfair bias and false positives/negatives results in disease detection or related interventions or treatment recommendations, notably where the user will have the possibility to interact with the AIS settings. Prospective randomised clinical studies should be privileged to generate sound evidence about the AIS performance. Context-specific testing may be necessary to ensure for example that an AIS developed in high income countries will be appropriate for generating public health recommendations for low- and middle-income countries or needs specific training or adjustments to avoid risks of misleading users [33] (p. 109). Then, the system could be scaled up and diffused. AIS providers must be able to perform post-marketing monitoring of their device, in collaboration with deployers and users for engaging in a quality management process.

Ethical tensions could arise between the principles of transparency and accuracy. As it is implicitly suggested by the UNESCO, if such a case occurs in the medical context, and we could add in public health contexts, accuracy should prevail where sufficient experimental data support the use of the AIS as the best option to achieve a clinical, or a public health goal [33] (p. 107). A high level of accuracy, of predictive value, and appropriately justified levels and ratio of sensitivity and specificity, based on the context and intended use of the system [91] is particularly important for AIS used in outbreak responses where the outputs will base decision-making on mandatory restrictions to individual rights and freedoms for public health reasons. This must be documented [92] and shared for further research and potential standardisation. Nevertheless, this trade-off should remain exceptional and justified by a particularly pressing medical or public health challenge.

**7. Environmental protection and sustainability** are a major challenge regarding AIS [32] (Principle 31, Policy Area 5) and a major public health concern in light of climatic changes and the global rarefaction and shortage of resources [93]. The development and use of AIS require to diminish negative impacts on the environment in order not to grieve the benefits of the technology. AI hardware and software necessitate manufacturing and functional resources such as big data centres and computational power consuming essential resources such as water, metals, energy, and generates carbon footprint. This calls for innovative models for designing, manufacturing, training and maintaining AIS by using energy-efficient algorithms and infrastructures to ensure sustainability in the long-run. This includes engaging in responsible purchasing throughout the AI value chain and contributing to the circular economy [94]. For instance, the AI’s increasing energy needs are especially criticised regarding electricity consumption that comes from non-renewable sources. The energy needs of AIS vary widely depending on the type of model and how it’s used [95]. The environmental cost of AI, in particular of generative AI [95,96] (e.g., generating one image taking as much as energy as fully charging a smartphone [97]), and the related social responsibility of AIS providers in the deployment of such systems, requires further developments and adoption of specific regulation at international level, standard assessment metrics [98] and accountability measures considering direct and indirect environmental, positive and negative impact, throughout AIS lifecycle [99]. Researchers emphasise the need for better data and more transparency from tech firms to accurately assess and manage AI’s energy consumption as the technology continues to evolve [95]. Practical tools for designers and users should be developed at international level. For instance, in France, the Agence du Numérique en Santé (ANS) is proposing an open tool named “EcoScore” to measure the environmental footprint of digital health applications [100]. Such a scoring is made mandatory for referencing in the e-health apps catalogue maintained by the ANS and available to patients and professionals through the e-health record “Mon Espace Santé” [101]. Nevertheless, this tool is limited as it only evaluates the impact based on effects on clients’ devices [102]. The high level of demand, potentials and expectations around AI requires a higher attention on resolving concerns on the environmental impact of this technology before considering the sole profits extracted from commercialisation. From an ethical point of view, this calls to adopt a one health approach [103,104,105] and to support a frugal innovation [106,107] approach to emerging technologies such as AI in order to design a code of conduct and potential trade-offs equilibrating and refining the human-centric approach to AI. Ongoing research in modal compression or quantisation could provide solutions to preserve both AIS performance and decrease energy consumption. New ethical principles such as Design for Disassembly and Design for Recycling for AI hardware could be promoted, backed by hard law [108]. In terms of ethical trade-offs, some researchers argue that the benefits of AI in areas like climate modelling and energy grid optimisation could outweigh its energy costs [94].

**8. AI safety** principle calls for preventing harms and address both short-term and long-term effects of AIS uses [32] (Principles, point 27). If it requires that the user’s health be not endangered at the time of use through safety mechanisms, it also calls for longitudinal research on the health effects of AI and for regulation of potential harms that might arise from AI use, including where users are not trained public health professionals. The UNESCO recommends that Member States establish “research on the effects and regulation of potential harms to mental health related to AI systems, such as higher degrees of depression, anxiety, social isolation, developing addiction, trafficking, radicalisation and misinformation, among others”. In addition, they should “develop guidelines on human-robot interactions and their impact on human-human relationships, based on research […] with special attention to the mental and physical health of human beings”, in particular children and youth, and align them with human values and human rights [32] (Policy Area 11, p. 37). This includes studying the long-term effect of AIS on human cognition capacities and to verify the validity of the classical standpoint stating that AI enhances human cognition without replacing it. Indeed, in public health, AIS are both part of the means to address global health challenges and a source of new important health challenges for humanity related to the specific characteristics of AIS.

**9. Stakeholder involvement and co-creation** is emphasised as a gold standard in responsible innovation. For example, performing ethics-by-design and related assessments necessitate to pool an interdisciplinary team with key stakeholders including engineers and technical experts but also scientists, public health professionals, ethicists, legal support teams, philosophers and other experts as necessary [34] (point 1.5), [32] (points 110, 130). They should be carefully selected with regard to the intended purpose and context of use of the AIS. They should involve external and independent persons. This team shall also include the people or representatives of the people that will act as end-users or that will be affected by the use of an AIS. In short, interdisciplinary teams should be a good variety of competent or relevant people who have a particular knowledge of the field in which the AIS is or is intended to be deployed and that can collaborate for improving the system. All shall be able to express their experiences and opinions and to be protected with regard to their freedom of expression. The leader of the assessment will have a crucial role in providing necessary information about the boundaries of the assessment and any other information or access to documentation relevant to perform fair and fine assessment. In this regard, use-cases are very useful to identify or present issues and collectively envisage efficient palliative measures to be implemented and whose result will need to be evidence-based. Stakeholders and the larger public should be transparently informed about the result of the consultation and co-creation process, of potential trade-offs made and remaining issues. Obviously, this is skills- and resource-consuming, but very much needed to ensure long-term confidence and adhesion of users. Stakeholder involvement must not stop after the putting into the market of the AIS. Feedback loops shall be set up and effective throughout the lifecycle of the AIS to feed efforts of constant quality improvement of the provider. Stakeholders involvement also calls for safeguards against data colonialism for ensuring appropriate engagement and benefit-sharing strategies [33] (pp. 39–40). In a broader concept of societal well-being, stakeholders’ involvement should be conceived as a way to promote and protect democracy [34] (point 1.6).

## 4. Discussion

### 4.1. A New Momentum for Conciliating Individual and Collective Ethical Approaches in the Perspective of International Public Health Actions

AI developments are the occasion to reaffirm and eventually develop new fundamental ethics principles in public health at international level while strengthening capacities to face current and emerging global public health challenges. But it is useful to remember that in the context of public health ethics, high-level principles and tools alone are not solving substantive ethical issues nor regulatory tensions that can exist in particular contexts. Therefore, it is important to reinstate the need to have evidence-based AIS in public health that will be tailored to the needs and will be affordable and sustainable.

Moreover, it calls to educate the different publics to AI ethics and AIS uses in public health, including policy-makers and health workers [109], with a neutral and pragmatic approach, in the long run. Educational programs should include training and responsibilisation of mass media regarding integrity in scientific and technological communication. Investments in education to improve AI literacy of the population is crucial to avoid AIS misuses, unfounded projections, fears, to maximise adhesion and diminish risks of controversies which could damage public health interventions, notably in times of crisis (e.g., misunderstanding about vaccines during the COVID-19 pandemics). AIS, even if developed according to ethics-by-design, will only be useful if the population adheres to them, if they are legally framed, and if users are properly trained. In this regard, the populational value of individually acquired or generated digital data for broader public health purposes seems insufficiently highlighted to the public, nor ethically or regulatory grounded in a comprehensive manner. We optimistically propose that this public health value of digital data be not only thought of in all health-related AIS but as an integrated optional extension of any digital device, as a new collective paradigm, to contribute, via the data, to the resolution of the pressing public health challenges facing humanity. Supporting ethical values and contexts of activation must be identified and explained to the data subjects. In particular the values of solidarity, of public interest, of common good and human rights, should base such an ambitious approach. The provision of information on guarantees ensured that answer to ethical and regulatory constraints described above could be provided generally, and specified further in use cases. This proposal would tend to systematically integrate an informational public health support functionality in digital devices and platforms that would be piloted by public health institutions. Building on the lessons learned during COVID-19 crisis on technical potential and acceptability, several options could be envisaged in the design of such a public health support functionality in collaboration with concerned private actors. For example, it could range from specific support to the spreading of information campaigns explaining the value and rationale of individual data contribution to the training of AI algorithms and global public health research, its ethical and regulatory bases and guarantees for individual rights to facilitate users’ engagement, to the design and integration of a specific public AI software able to prepare and anonymise data for specific public health uses, on-demand. Because this latter option could be seen as very intrusive, the software activation should only be in the hands of public institutions, optional and transparent to users, justified by a legitimate public health need or event and framed by law. This functionality should be paired with adapted human oversight and mechanisms to enable individuals’ autonomy in data sharing.

### 4.2. AI Development as an Opportunity to Strenghten Translational Collaborative Research by Improving Innovation Practices and Regulatory Environment

Today, AIS in health seem first considered as supporting tools for targeted clinical applications [110,111] and personalised healthcare and prevention at individual level, for precision medicine development. This can be seen as a first step to achieve an appropriate technology readiness level (whose criteria are still to be fixed) in a regulated environment that integrates ethics-by-design [112] before scaling-up for epidemiological uses.

Even though most of the AIS are conceived for clinal care, there are only few innovations that are finally deployed and used in real-world and on the long run. This translational bottleneck could be solved by adopting pragmatic and inclusive design approaches involving public health professionals. Researchers proposed an operational guide to facilitate the transition of academic AIS to operational tool that can be instrumental for the design of deployable public health innovations [108]. This guide is in line with the ethics-by-design approach and clearly identifies stakeholders, responsibilities, key issues, prerequisites and solutions to prepare deployment and build deployable tools that improve chances of efficient operationalisation. In this dynamic, the public health contribution of clinical AIS design and deployment should not be disregarded but integrated in the process. These aspects call for adapting standards and health technology assessments methods as well as requirements applied to medical devices as some AIS could legally be qualified as such.

An essential pre-requisite to this ambitious goal is to build federated data infrastructures and standardised models to maintain sufficient data quality and security to develop trustworthy public health AIS at international level. Public–private partnerships should be privileged to develop these capacities, to avoid the development of fully privatised systems and join forces to create valuable and auditable AIS for public health uses that could be more easily adopted, economically affordable and tailored to specific needs of certain populations or geographical areas. Regulatory initiatives for facilitating health data quality management and open access for public health research purposes, such as with the EHDSR, could build on more transparent and direct connections between individuals and collective entities based on ICTs for ensuring ethical and lawful AIS developments and uses, notably regarding personal data processing. The States and public health authorities should (re)gain a stronger gate-keeper role in ensuring and organising responsible health data sharing for innovation, while maintaining contacts with the population and with individuals that can exercise their rights where personal data or privacy issues arise. Governments should impose new standards to innovators and new means for States and individual actions [32] (point 109) equilibrating powers with big private technology companies [113], in the respect of human rights.

### 4.3. The Necessity of Strenghtening International Monitoring, of Elaborating Specific Guidelines and Developing Policies to Push Forward Ethics in AI for Public Health

In a context of a global technological race to AI, more or less honest advertising campaigns and potential hype from the technology providers are raising specific ethical issues in communication and call for a cautious approach. This may lead to reconsider the added value of bringing AI into the public health sphere and to refrain from integrating immature technologies into the domain. Research on AIS applications in the field must be promoted to facilitate evidence-basis adoption. As an example, during the COVID-19 pandemic, while many AIS allowed to better prevent, detect and diagnose the disease, a lot of hype and hope around AI took place. Nevertheless, studies such as the one from the French CNIL LINC [114] showed that AI only played a subsidiary role in the fight against the pandemic. Therefore, beyond the hype, it is important to bear in mind important caveats and limitations of AI in these exceptional contexts. First, AI-enabled diagnostics are still in their infancy and remain closely tied to domains for which large amounts of annotated data are available, such as medical imaging. Second, AI is based solely on previously observed behaviour in training data. But in the context of COVID-19, exploitable data from previous large pandemics were inexistent or inappropriate with regard to the breadth of this one. Data had to be collected from the ground and could not benefit from historical epidemiological datasets to perform accurate predictions. This can lead to algorithmic biases, especially in times of health crisis when risks are exacerbated. Some research is already pointing to the presence of bias in COVID-19 diagnostic aid systems using AI. Therefore, the study from the CNIL LINC concludes to a moderate capacity of current AIS to cope with the so-called “black swan” events [115] which are highly improbable and unpredictable based on available historical data while they have a massive impact, extreme consequences and can be rationalised and explained afterwards.

In reality, the AI used today is fairly weak and has not enabled the most technologically advanced countries to better manage the pandemic. Also, this specific use-case exacerbated a pre-existing risk of being tempted by technological solutionism [116] while other challenges related to health systems organisation and capacities are critical to predict and palliate such situations. Nevertheless, using digital means during black swan events allows to collect massive data that will be used as informational resources for innovations in public health technologies that could be much more useful where similar events will occur.

Therefore, there is a global need for States to reinforce public monitoring of the self-government practices and ethical claims from private actors [33] (point 9.3) in public health AIS, whether they are innovators, deployers or big data providers, in order to detect any ethical misconduct and act consequently. The same goes with regard to public sector institutions using public health AIS [33] (point 9.4). In this regard, it seems equally important to provide specific guidance to research ethics committees that will be engaged in the approval or follow-up of public health projects involving AIS [e.g., [117]].

Specific ethical guidelines supporting responsible public health AIS development and use in scientific research, in public policy-making, and for interventions shall be further developed at international level and reinforced by hard law and connected technical standards. Despite the political and financial vicissitudes affecting science and collaborations [118], the WHO shall have a particular role in further detailing ethics principles for public health AI and fostering adhesion to tailored international standards that could lead to specific certification processes. Quality of service indicators [34] (p. 22) could also be usefully developed at global level for providing a baseline understanding about an AIS trustworthiness, based on the respect of the public health AI ethics principles, for informing the public health professionals community. Because high-level ethical principles are often insufficient to solve practical issues, the development of a public health AI ethics based on procedural values should be supported and further elaborated to operationalise ethics [119], such as with the Accountability for Reasonableness model [120], serving institutional accountable moral decision-making process [11]. Finally, in collaboration with governments, the WHO should be able to monitor the respect of human rights and ethical values in order to detect, investigate and prevent ethics dumping, ethics shopping, ethics bluewashing, ethics shirking or counterproductive lobbying [121] (chap. 5).

## 5. Conclusions

We note a global convergence of ethical principles to consider in responsible AI innovation at international level. These principles align with the main principles of public health ethics and digital health ethics [e.g., [122]] and are helpful in fostering human rights protection and developments provided that they are accompanied by support infrastructures and implementation tools. If the global approach to public health AI ethics serves alignment in technological developments that should foster appropriateness and adoption of AI technologies, it appears that both the existing principles and tools deserve more practical and focused guidance on public health use-case at international level. Nowadays, clinical developments should go hand-in-hand with public health ones and maximise the translational value of technologies. The public health dimension should be seen as an integrated part of digital tools and be part of educational programs for citizens, innovators and health professionals, to further sensitise the different publics to the potential of digital data to drive innovations in the public health interest, without resulting in a general obligation to participate. As we are still in the early stages of AIS applications in clinical and public health settings, there is a real opportunity to implement a paradigm shift towards more inclusive innovation in the field. This could base new ethical and legal developments and communication efforts based on the principles of solidarity, democracy and human rights. New technical standards supporting integrated functionalities for public health contributions could be developed in most of the digital tools and AI applications generating data. The EU digital data legislations can be seen as paving the way towards such a model at international level which, based on prior ethical works (e.g., the AIA and the HLEG Guidelines on Trustworthy AI) and a risk-based approach, elaborated mandatory rules allowing to equilibrate data protection and responsible data access with AI innovation needs. Even if the current EU laws such as the AIA, the GDPR, the DGA and EHDSR are still containing gaps to explain the translational process of ethical principles into legal provision, they build procedures for data access and innovation in the public interest that shall serve public health promotion and interventions. Importantly, it includes avenues for developing operational ethical decision-making processes based on procedure such as self-assessments (e.g., DPIA, FRIA and collective human AI oversight mechanisms) intending to concretise ethical principles. Because regulations on AI are emerging around the world [123], the study of the role of law in enabling or impairing public health improvements will also deserve further attention in the future, and legal epidemiology [124] studies will need to be supported.

## Data Availability

No new data were created or analyzed in this study. Data sharing is not applicable to this article.

## Abbreviations

The following abbreviations are used in this manuscript:

HUDERIA	Human rights, democracy and rule of law impact assessment
COVID-19	Corona virus disease, SARS-CoV-2 virus
UNESCO	United Nations Educational, Scientific and Cultural Organisation
CIOMS	Council for International Organisations on Medical Sciences
EHDSR	European health data space regulation
OSAID	Open source artificial intelligence definition
Art.	Article
Arts	Articles
C2PA	Coalition for content provenance and authenticity
CNIL	Commission nationale pour l’informatique et les libertés
EHDS	European health data space
EOSC	European open science cloud
FAIR	Findable, available, interoperable, reusable
FRIA	Fundamental right impact assessment
GDPR	General data protection regulation
HDAB	Health data access body
HLEG	High level expert group on AI
LINC	Laboratoire d’innovation numérique de la CNIL
LMMs	Large multimodal models
SNDS	Système national des données de santé
Rec.	Recital
AIS	Artificial intelligence system(s)
AIA	Artificial intelligence act
ANS	Agence du numérique en santé
DGA	Data governance act
HDH	Health data hub
ICT	Information and communication technology
IHR	International health regulations
MDR	Medical device regulations
NLP	Natural language processing
RAM	Readiness assessment methodology
WHO	World health organisation
AI	Artificial intelligence
CR	Content credentials
MS	Member State(s)
EU	European Union

## Appendix A

### Appendix A.1

**Table A1:** Overview of the main AIS applications in public health.

Application Purposes	Benefits
Improve medical diagnosis	- By facilitating the analysis and management of medical records using natural language processing (NLP); - By facilitating the analysis of biological and medical imaging tests to improve characterisation of abnormalities or diseases and to design patterns indicative of diseases or medical conditions [125] that will serve public health research; - By facilitating detection of potential errors in medical prescriptions or surgical procedures thanks to in-depth data analysis.
Improve diseases prediction and prevention	- By developing and/or using predictive models [126] to predict, detect and estimate at-risk populations and adapting screening strategies based on individual medical data, patient history or other types of relevant data; - By developing models and applications for optimising patient prognosis and for guiding personalised treatment strategies based on data crossing at individual and populational levels [127].
Improve epidemiological surveillance and early interventions [128]	- By developing models allowing early detection of infectious disease outbreaks by analysing public health data, social media and other sources to better understand population health behaviour, identify abnormal trends and risk factors for chronic or emerging diseases and develop targeted prevention and intervention strategies [129,130,131]; - By developing AI-based applications, such as applications on mobile phones or connected objects, for monitoring patients or medicinal products (e.g., for reporting more efficiently adverse drug effects through dedicated chatbots), and taking on-time preventive action [33]; - By combining clinical decision support systems [132] and patient self-management tools for better population health management [133].
Improve management of healthcare resources [134]	- By forecasting healthcare demand and efficient allocation of medical resources, such as hospital beds, medical staff and medical supplies; - By allowing smart orientation of patients based on geographical, technical, economic, environmental and human resources constraints leading to best taking in charge and patients flow management; - By providing more efficient tools for e-health and telemedicine to professionals and patient; - By facilitating healthcare administration, diminishing bureaucracy, errors, and enhancing coding of medical information in medical records and facilitating data interoperability for reuses in public health purposes [110] (point 2.5).
Accelerate biomedical research	- By optimising the organisation of clinical trials through data-driven processes [135], in terms of design, patient recruitment and enrolment, execution and management; - By facilitating bibliographic research; - By facilitating the analysis of vast sets of genomic and molecular data to identify new therapeutic targets and accelerate the drug discovery process.
Facilitate population health education and awareness [136,137]	- By developing reliable AIS such as chatbots [138,139] and virtual assistants to provide accurate public health information and personalised health or medical advice and support (e.g., in mental health) to the population; - By developing specific educational systems for training public health professionals based on simulated situations at population or individual level; - By helping in the design of best adapted educational programs and materials fitting the needs of the concerned populations.
Contribute to and evaluate the effectiveness of public health policies [140]	- By facilitating analysis of data about health practices, gaps, new challenges and avenues for innovative improvements of existing strategies; - By facilitating the evaluation of the cost-effectiveness of public health interventions in a given health context, before, during and after the intervention, for decision-making purposes or for envisaging new types of interventions.

### Appendix A.2

**Table A2:** Risks associated with AIS development and use in public health.

Risks	Impacts in Public Health
Manipulation and biased interpretation of health data	The use of certain predictive or generative AI models in public health entails risks of manipulating users or biasing health data to support certain political strategies. This concern is amplified by the lack of transparency in AI algorithms, which makes it difficult to understand the mechanisms behind predictions and decisions, particularly where deep learning techniques are used. Poor data management and bias control could lead to unfair or ineffective public health decisions, particularly affecting vulnerable populations [141]. Generative AI models raise additional challenges related to potential hallucinations amplifying bias and producing false results while answering questions [142].
Stigmatisation of individuals at risk	AIS models may amplify risks of stigmatisation of individuals identified by AI as being at high risk of developing chronic diseases, in particular if training data are biased and contain societal misrepresentations. AI applications in public health may use sensitive personal data and algorithms that can reinforce existing inequalities, creating discrimination based on medical history or specific ethnical, geographic or demographic characteristics. This could affect access to health insurance [143] or specific treatments, with potentially lasting effects on the health of marginalised populations.
Lack of privacy and data security	The collection and analysis of health data, including behavioural and medical data, pose privacy risks. In the EU, personal data fall under the GDPR duties and respect for fundamental rights. Any public health AIS should consider privacy-by-design throughout the data lifecycle. Privacy breaches and the possibility of cyberattacks on AIS and health databases are ongoing concerns [141] that can expose personal data to unintended uses compromising patients’ rights and trust in both the technology and public health activities [144].
Poor analytical reliability and risks of false or misleading alarms	AI, while effective for the early detection of disease or the prediction of epidemiological trends, is not free from error. Systems can generate false alarms or fail to detect medical errors, which could lead to inappropriate actions or failures in disease prevention and management. These problems are exacerbated by the lack of robustness and transparency of the algorithms [47], increasing concerns about their widespread use in public health.
Excessive surveillance and infringement of fundamental rights	The use of AIS for constant monitoring of public health raises questions about possible excessive surveillance and overly intrusive government control. Automating public health processes could limit the independence of AI analyses and raise issues about the ethics of decisions based on these systems [145]. It may also create concerns about the use of data for purposes other than those originally intended, such as commercial profit or political surveillance [146].
Inequitable access to quality care or preventive measures	As AIS rely on internet and modern data processing infrastructures coverage, they will not be uniformly deployed in all countries, nor in all healthcare systems, and some institutions may not have the resources to integrate these technologies. This disparity in access to medical AI could widen inequalities in care, particularly in disadvantaged regions [147] and create disadvantages for public health outcomes. Digital exclusion of vulnerable populations, such as the elderly or individuals with low connectivity, is also a concern.
Loss of human control, dehumanisation of care and reductionism	The risk of over-reliance on AIS for public health intervention design and implementation could lead to a dehumanisation of care, with less direct contact between public health professionals, carers and patients. AI could be perceived as an intermediary that reduces the importance of social skills such as collaboration, empathy and compassion, in particular in the carer-patient relationship. Moreover, the increasing automation of tasks could transform medicine into a purely technical process, distancing healthcare professionals from their traditional clinical expertise [148] and increasing reductionism perceptions of populations, health determinants, affecting then the moral acceptability of public health interventions based on AIS and aggregated data. Human oversight of AIS processes, autonomy of the various users and concerned persons’ responsibilities in case of damage due to erroneous results or misuse of AIS are crucial topics to address for acquiring and keeping confidence in those systems, in particular in critical public health decision-making processes.
